# DHEA inhibits regeneration in *Lumbriculus variegatus*

**DOI:** 10.17912/micropub.biology.001898

**Published:** 2025-12-08

**Authors:** Erin Frank, Iris Pardue, Ifti Rahman, Emily Banthin, Kathy Gillen

**Affiliations:** 1 Molecular Biology, Kenyon College, Gambier, Ohio, United States; 2 Biology, Kenyon College, Gambier, Ohio, United States; 3 Comprehensive Cancer Center, Radiation Oncology, The Ohio State University, Columbus, Ohio, United States

## Abstract

Some organisms can regrow severed body parts. In vertebrates, the anabolic pentose phosphate pathway (PPP) aids regeneration, but we do not know if invertebrates require the PPP for regeneration. To test this, we attempted to inhibit the PPP rate limiting enzyme, glucose-6-phosphate dehydrogenase (G6PD), with the steroid dehydroepiandrosterone (DHEA), using the regenerative annelid *Lumbriculus variegatus* as our model organism. DHEA inhibited *Lumbriculus* regeneration, a result also observed in tadpole tails, which may suggest conserved regenerative mechanisms in vertebrates and invertebrates. Notably, we did not observe G6PD inhibition with DHEA treatment, suggesting that DHEA may be working through a G6PD-independent mechanism.&nbsp;

**Figure 1. Dehydroepiandrosterone (DHEA) inhibits Lumbriculus variegatus regeneration, but likely not through the pentose phosphate pathway:**
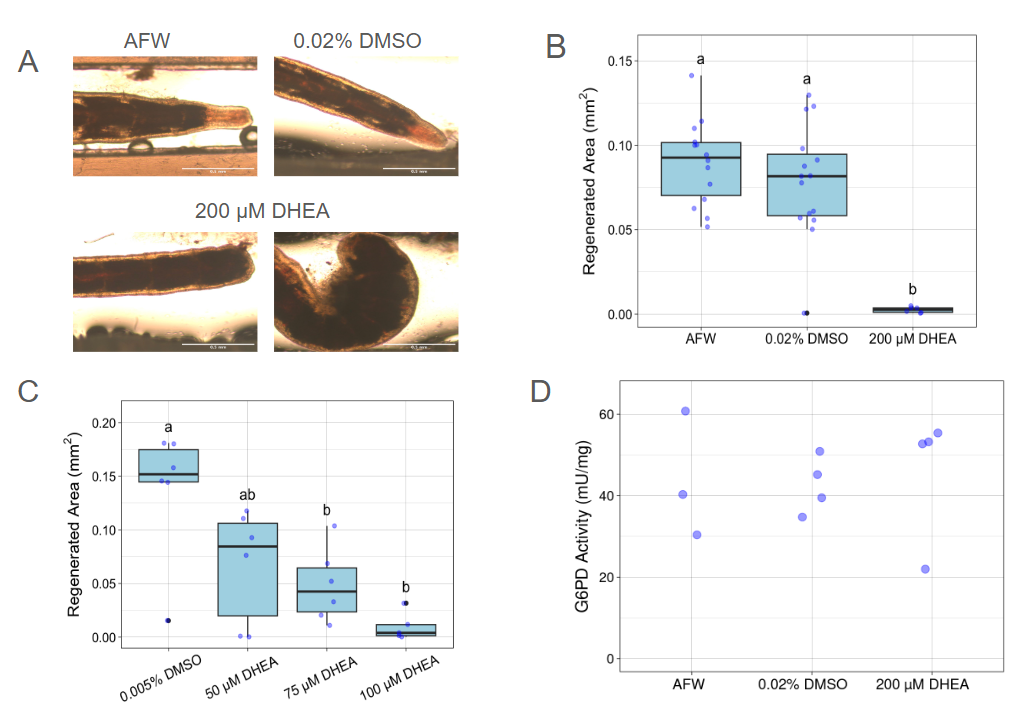
**(A)** Representative images of worms exposed to artificial freshwater (AFW), 0.02% dimethyl sulfoxide (DMSO) vehicle control, or 200 µM DHEA for 24 h, cut, and then placed back in their respective solutions for 4 days prior to imaging. Newly regenerated areas have less pigmentation. The scale bar is 0.5 mm. **(B)** Worms (n=15 per condition) were exposed to AFW, DMSO, or 200 µM DHEA as described for panel A. Sample size for image analysis: AFW n=14; DMSO n=15; DHEA n=6.&nbsp; Each blue dot is the average of three blind measurements of an individual worm; outliers are also shown in black. Treatments with different letters were significantly different from each other at p ≤ 0.001&nbsp; (ANOVA with Tukey, df=2, F=22.91, p=0.006). **(C)** DHEA dose-response. DMSO control, 50 µM, and 75 µM DHEA (n=6); 100 µM DHEA (n=5). Worms were pre-treated for 28 h, cut, and replaced in respective solutions for 5 more days prior to imaging. Each blue dot is the average of three blind measurements of an individual worm; outliers are also shown in black. Treatments with different letters were significantly different from each other&nbsp; at p ≤ 0.05 (ANOVA followed by Tukey HSD, df=3, F=7.68, p ≤ 0.01). **(D)** G6PD activity (mUnits/mg) was assayed in whole worms incubated in AFW (n=3), 0.02% DMSO vehicle control (n=4), or 200 µM DHEA (n=4) for 24 hrs. Biological replicates consisted of tissue homogenates made from two whole worms. G6PD activity was normalized to total protein concentration using a BCA assay. No differences in G6PD activity were detected between DHEA treated worms and vehicle control or AFW control (Kruskal-Wallis, p ≥ 0.05).

## Description

Many species can regenerate large portions of tissue lost upon injury. Understanding the mechanisms driving such regeneration may provide insight into fields such as regenerative medicine or diseases with highly proliferative tissues like cancer. One mechanism that has been explored is the upregulation of the pentose phosphate pathway (PPP). The PPP is an alternative pathway to glycolysis and produces molecules essential to cell proliferation such as NADPH, a key precursor for fatty acid synthesis, and ribose-5-phosphate, an important precursor for nucleotide synthesis (TeSlaa et al., 2023). Supporting the notion that the PPP is important in highly proliferative cells is the observation that many cancerous cells often exhibit the Warburg effect whereby the PPP is upregulated (Vander Heiden et al., 2009).

The PPP was recently shown to be important in *Xenopus* regeneration (Patel et al., 2022) where after tadpole tail amputation, the PPP rate limiting enzyme, glucose-6-phosphate dehydrogenase (G6PD), was upregulated. Furthermore, two G6PD inhibitors, G6PDi and dehydroepiandrosterone (DHEA), reduced tail regrowth. While the PPP aids vertebrate regeneration, we do not know if it is required for invertebrate regeneration; to investigate this, we used the invertebrate *Lumbriculus variegatus* , a freshwater annelid long used as a model organism in regeneration research (Martinez Acosta et al., 2021).&nbsp;

To investigate the role of the PPP in invertebrate regeneration, we tested the effects of DHEA on *L. variegatus* regeneration. Worms were pretreated with 200 μM DHEA for 1 day, followed by continued DHEA exposure for 4 days post-amputation. Normal regeneration was observed in DMSO vehicle control exposed worms ( Figure 1A and B). DHEA treated worms experienced high mortality, with only 6 out of 15 still alive 4 days post-amputation. Two of the six surviving DHEA treated worms were bloated ( Figure 1A ) and none of the survivors regenerated ( Figure 1B, ANOVA, Tukey HSD, p ≤ 0.001). We also examined if lower concentrations of DHEA could elicit the same effect. In a dose-response experiment, 100 μM DHEA stopped regeneration nearly as effectively as 200 μM DHEA, while 75 μM DHEA reduced median regeneration by 72% compared to the DMSO control ( Figure 1C, ANOVA, Tukey HSD, p=0.015). Treatment with 50 μM DHEA trended towards reduced regeneration but did not reach statistical difference from the control group. None of these lower DHEA treatments caused the high mortality seen with the 200 μM DHEA treatment. We further investigated if the reduced regeneration was due to DHEA inhibiting G6PD. DHEA treated worms showed no significant reduction in G6PD activity when compared to DMSO vehicle control and artificial fresh water (AFW) treated worms ( Figure 1D, Kruskal-Wallis, p ≥ 0.05).&nbsp;

Our lack of evidence for G6PD inhibition by DHEA is unsurprising because while DHEA has been characterized as an inhibitor of G6PD since the 1960s, it was suggested DHEA may only inhibit mammalian G6PD (Marks and Banks 1960). Additionally, while DHEA robustly inhibited human G6PD in vitro, inhibition in vivo was minimal (Ghergurovich et al., 2020). Of note, DHEA is an unusual inhibitor as it has been reported to inhibit mammalian G6PD uncompetitively, binding exclusively to the enzyme-substrate complex (Raineri and Levy 1970; Gordon et al., 1995). Since the G6PD enzyme-substrate complex in *Lumbriculus* remains uncharacterized, it is possible that the annelid complex differs enough from the mammalian complex to not allow G6PD inhibition by DHEA. Another possibility that might explain our lack of G6PD inhibition by DHEA is that in vivo DHEA may be converted to another steroid and not all steroids inhibit G6PD (Raineri and Levy 1970).

If the steroid DHEA is not blocking G6PD activity within *Lumbriculus* , the compound or one of its metabolites may be inhibiting *Lumbriculus* regeneration through binding steroid hormone receptors. Steroid hormones are known to affect proliferation of cells and even regeneration of deer antlers. DHEA likely inhibits proliferation of human breast cancer cells through binding androgen receptors (ARs) (Boccuzzi et al., 1993) and this may be how the compound inhibits regeneration in *Lumbriculus* . Elsewhere in the animal kingdom, antler regeneration in male deer is governed by tightly controlled fluctuations in testosterone levels and exogenous androgen treatment caused female deer to grow antlers (Li et al., 2003). However, unlike high-affinity estrogen receptors (ERs) which have been identified in the marine annelids *Capitella telata* and *Platynereis dumerilii* , (Keay and Thornton 2009), the existence of active ARs in annelids remains unestablished. As such, it is challenging to propose a mechanism of action for DHEA in *Lumbriculus* involving ARs. As for ERs, DHEA may have transcriptional impact in *Lumbriculus* as the chemical is thought to activate mammalian ERs (Webb et al., 2006). Nevertheless, the extent to which such steroid mechanisms are conserved across vertebrates and invertebrates is still unclear. Future experiments might use steroid hormone receptor agonists and antagonists to test if the effects of DHEA on regeneration are dependent on hormone receptor pathways.

&nbsp;Our finding that DHEA inhibits regeneration in *L. variegatus* has at least two implications. First, it argues for limiting steroid hormone pollution of waterways. Pharmaceutical and agricultural steroid hormones are found in the environment and treated wastewater (Almazrouei et al., 2023) and even low concentrations of some steroids can harm aquatic animals such as fish and amphibians (Ojoghoro et al., 2021). Furthermore, current research suggests that aquatic annelids may take up steroids from the environment (Priscilla et al., 2023). Therefore, the inhibition of regeneration in DHEA exposed worms may negatively impact wild populations of *Lumbriculus* , which, as detritivores, serve key roles in the food chain (Martinez-Acosta and Zoran 2015; Majlesi et al., 2025). Second, similar to the results seen with reactive oxygen species inhibitors (Love et al., 2013; Beinart and Gillen 2025), DHEA inhibits regeneration in both frogs and annelids, suggesting that conserved molecular mechanisms may underlie regeneration in both vertebrates and invertebrates, thus strengthening the utility of *L. variegatus* as a model organism for regeneration research.

## Methods

**Organisms and Materials:** &nbsp; *Lumbriculus variegatu* s were purchased from Carolina Biological Supply company (catalog # 141720). We previously determined that Carolina Biological worms are Clade I polyploid worms (Fischer et al., 2022). Worms were maintained in artificial freshwater (AFW, 0.5 g of Instant Ocean Sea Salt/L of deionized water) at 18 °C. Twice weekly, worms were fed 2-3 pellets of Frog and Tadpole Bites (Pisces Pros, upc 788459100303). Water was changed at each feeding. One molar dehydroepiandrosterone (DHEA, Sigma-Aldrich 252805) stock solutions were prepared in dimethyl sulfoxide (DMSO, Sigma-Aldrich D8418). A protease inhibitor (PI, Thermo Scientific #88666) was dissolved in 1x phosphate buffered saline (PBS, Fisher BP399-1); this PBS-PI was used to make tissue homogenates. G6PD activity was measured as per assay kit (abcam, ab176722) and normalized using total protein concentration measured by BCA assay kit (ThermoFisher Pierce Protein Biology, #23227).&nbsp;

**DHEA treatments** : DMSO vehicle control and DHEA solutions were prepared by diluting 0.2% DMSO and 1 M DHEA stock solutions respectively in AFW. In Figure 1A and B, worms previously starved for 48 h were placed into individual wells of a 24-well tissue culture plate containing AFW, 0.02% DMSO, or 200 µM DHEA (one worm per well, n=15 per treatment) and incubated for 24 h prior to amputation. Following excision roughly 15 segments from the head, anterior ends of worms were placed back in their respective solutions and tracked for posterior regeneration. 96 h post-amputation (hpa), worms were imaged for data analysis. In Figure 1C, whole worms were incubated in 50, 75, or 100 µM solutions of DHEA or a 0.005% DMSO vehicle control for 28 h prior to amputation. Worms were then cut ~12 segments from the head and their anterior ends placed back into their respective solutions to track posterior regeneration. 120 hpa, worms were imaged for data analysis.

**Microscopy and calculating regeneration area:** We obtained brightfield microscopy images on a Nikon Optiphot-2 microscope outfitted with a Zeiss Axiocam 503 color camera. Images from Zen 3.8 were imported into FIJI (Schindelin et al., 2012). Using the freehand tool, the newly regenerated area (the area with less pigmentation) was demarcated and its area recorded. Each area used in data analysis was the average of three blind measurements.&nbsp;

**G6PD Activity Assay:** Whole worms were incubated in either a 200 µM DHEA solution or a 0.02% DMSO vehicle control solution for 24 h, eight worms each. Six whole worms were also incubated in AFW for 24 hrs. After incubation, tissue homogenates were prepared using two whole worms for each biological replicate producing four biological replicates for DHEA and DMSO treated worms and three biological replicates for AFW treated worms. To homogenize tissue, worms were snap frozen in liquid nitrogen and then pulverized with a pestle in 1x PBS-PI and finally centrifuged at 4 °C for 8 min at 1,300g. A portion of the supernatant was removed to quantify total protein concentration with a BCA assay kit. To determine G6PD activity, 6 µL was diluted to 120 µL and assayed per kit instructions, with technical duplicates of all samples. The samples were read immediately and every 5 minutes for 2 h on a Molecular Devices SpectraMax Gemini EM with SoftMax Pro 7.0.3 software. G6PD activity was determined based on fluorometric changes across the linear phase of change (0 min and 55 min) and then normalized by total protein concentration.

**Statistical analysis** :&nbsp; To determine differences in regeneration areas we ran an ANOVA followed by Tukey HSD. Differences in G6PD activity were analysed by a Kruskal-Wallis test. All analyses were done in R-studio version 4.4.3. A p-value of ≤ 0.05 was considered significant.
